# ‘Autistic person’ or ‘person with autism’? Person-first language preference in Dutch adults with autism and parents

**DOI:** 10.1177/13623613221117914

**Published:** 2022-08-11

**Authors:** Riley Buijsman, Sander Begeer, Anke M Scheeren

**Affiliations:** Vrije Universiteit (VU) Amsterdam, The Netherlands

**Keywords:** adults, advocacy, autism, identity-first language, person-first language, terminology

## Abstract

The language used to refer to autism has been a topic of ongoing debate. Research in English-speaking countries indicated an overall preference for identity-first language (‘autistic person’) among autistic adults rather than person-first language (‘person with autism’). We examined terminology preference in Dutch autistic adults (*n* = 1026; 16–84 years; 57% women) and parents of autistic children (*n* = 286) via an online survey. A majority of self-reporting adults with autism (68.3%) and parents (82.5%) demonstrated a person-first language preference. A younger age, higher IQ and more autistic traits predicted a relatively stronger identity-first language preference in autistic adults. We conclude that language and culture may impact terminology preference of adults with autism and parents. For now, we advocate to use a mix of person-first language and identity-first language in academic papers to cover the full range of preferences.

**Lay abstract:**

There are different words to describe people with an autism diagnosis. For instance, we can put the person before autism (e.g. ‘person with autism’), or we can put autism before the person (e.g. ‘autistic person’). Previous research showed that autistic adults in English-speaking countries generally liked it better when autism is placed before the person. Yet, people also greatly differ in the words they like and dislike. In this study, we examined word preference in Dutch autistic adults (*n* = 1026; 16–84 years; 57% women) and parents of autistic children (*n* = 286). Via an online questionnaire, we asked our participants to select one term for autistic people that they liked best. The results showed that most adults with autism (68.3%) and parents (82.5%) preferred to put the person before autism. Younger adults, with a higher intelligence, and with more autistic traits, were a bit more likely to put autism before the person. We conclude that there are large differences in the words that people prefer. Because we found different results in our Dutch participants compared to participants in English-speaking countries, we think that the Dutch language or culture may also play a role in word preference. For now, we advise autism researchers to use both person-first and autism-first language.

The terminology used in reference to persons diagnosed with autism spectrum disorder (ASD) has received increasing attention (Bury et al., 2020). A distinction can be made between terminology which places person before identifier, termed person-first language (PFL), for example, ‘person with autism’, and terminology which places identifier before person, termed identity-first language (IFL), for example ‘autistic person’. The use of PFL dominated autism research, but this convention is changing (Shakes & Cashin, 2019). In 2010, the American Psychological Association (APA, 2010) recommended the use of PFL, because IFL supposedly ‘objectifies a person by her or his [*sic*] condition’ (p. 76). Yet, in 2020, this recommendation was changed, and the APA (2020) advised to use what is appropriate given the context, such as the preference of participants themselves. Similarly, the guidelines of this journal (*Autism*) include either option (Autism Terminology Guidelines, n.d.). In contrast, the Autism Self Advocacy Network (Brown, n.d.), or the journal *Autism in Adulthood*, use IFL exclusively. However, the preferences of the autism community, including autistic adults, parents and professionals, have thus far only been systematically examined in two English-speaking countries (United Kingdom and Australia). Therefore, it seems relevant to assess terminology preferences in a non-English-speaking country. Throughout this text, the terms ‘autistic’ and ‘with autism’ are used interchangeably as a clear standard is lacking.

The words we use to describe autism and people with autism reflect and shape the way we think about autism, and words may therefore (unintentionally) contribute to stigmatization. Stigmatization of autistic persons includes society ascribing stereotypical traits to persons on the autism spectrum and consequently defining them by their autism instead of perceiving every autistic person as a unique individual (Botha et al., 2020; Cage et al., 2019). On the one hand, it has been argued that PFL counters stigmatization as it emphasizes the humanity of someone rather than their autism (West et al., 2015). It literally puts autism behind the person, supposedly promoting the view that people with autism are individuals who cannot be defined solely by their autism. On the other hand, because positive attributes are more commonly placed before rather than after a noun in the English language (‘beautiful people’ rather than ‘people with beauty’) and PFL is often used to refer to (attributes of) disabled individuals (e.g. people with an intellectual disability; Gernsbacher, 2017), PFL may promote the idea of autism as (only) a disability or even something negative, a view that the neurodiversity movement strongly opposes to (Kapp et al. 2013). Thus, instead of reducing stigma, PFL may in fact maintain and induce stigma and ableism (i.e. the assumption that disabled people are inferior to nondisabled people; Bottema-Beutel et al., 2020). IFL, then, may be an empowering alternative that has indeed been preferred by many autism self-advocates on social media who share the view that autism is a difference but not necessarily a deficit (Sabatello, 2019; Shakes & Cashin, 2020; Thibault, 2014). In that same line, autistic adults who see autism as a vital and inseparable part of their identity are more likely to be IFL proponents (Botha et al., 2020; Bury et al., 2020; Kapp et al., 2013). It should be added, though, that PFL proponents may also view autism as an important facet of their identity (Bury et al., 2020).

There are only a few empirical studies on PFL/IFL preferences among the autistic and autism community. In a large-scale study in the United Kingdom, IFL, more so than PFL, was more often endorsed by autistic adults (*n* = 502; age range: 19–66+ years; 57% female) and parents of autistic people (*n* = 2207) compared to professionals such as teachers (*n* = 1109) (Kenny et al., 2016). Around 40% of the autistic adults endorsed the term ‘autistic’ to describe themselves (compared to 30% of the parents and 30% of the professionals), but only 18% endorsed ‘person with autism’ (compared to 14% of the parents and almost half of the professionals). A similar IFL preference was recently reported by Lei et al. (2021) among autistic self-advocates (*n* = 37; age and gender unknown) and family members or friends of an autistic person (*n* = 250). In an Australian study, ‘autistic’ and ‘person on the autism spectrum’ were preferred most by, respectively, 38% and 25% of autistic adults (*n* = 198; *M*_age_ = 35 years; age range: 18–71 years; 57% females) and were also rated as least offensive by, respectively, 43% and 18% (Bury et al., 2020). In the latter study, men and women showed a similar preference, but non-binary individuals had a stronger IFL preference compared to both men and women. Degree of autism traits was weakly and positively correlated with a preference for IFL. Hence, a preference for IFL or PFL depends on several individual factors.

Given the wide variety of term preferences between as well as within groups (e.g. autistic adults), Kenny et al. (2016) concluded there is not one singular correct way to describe autism. Although consensus might be difficult to achieve, it has been proposed to select terms on the basis of what the majority prefers, likely resulting in more IFL given the prior studies in the United Kingdom and Australia (Bottema-Beutel et al., 2020). Context (e.g. the audience) may further shape the preference for and fitness of terms (Mackelprang, 2010). For instance, Facebook groups targeted at parents of children with autism mostly use PFL, while groups targeted at autistic persons mostly use IFL (Abel et al., 2019). As yet, there have not been empirical reports on terminology preference of autistic adults and parents in a non-English-speaking country. Language could influence people’s preferred choice of words. For instance, a study among Spanish-speaking participants of an online course on inclusive education revealed a strong PFL preference (e.g. persona con autismo / persona autista; Garcia-Molina, 2019), but these participants were neither autistic nor did they have family members with autism. Moreover, different syntactic rules (e.g. placing the adjective after the noun) in the Spanish language compared to English may have contributed to this difference.

We examined autism terminology preference in a large sample of Dutch-speaking autistic adults and parents of children with autism. Following Kenny et al.’s (2016) findings, we expected that both autistic adults and parents of autistic children would prefer IFL over PFL. Furthermore, in a secondary explorative analysis, we assessed whether autism terminology preference varies depending on gender, age, degree of autism traits, intellectual ability and educational level.

## Method

### Participants

The sample consisted of 1026 adults with autism and 286 parents of autistic children. All participants reported a formal ASD diagnosis established by an independent qualified clinician (e.g. psychiatrist) in a professional setting (e.g. mental healthcare clinic). The age of autistic adults ranged from 16.28 to 84.18 years (*M* = 44.75, *SD* = 13.72). See Table 1 for an overview of all participant characteristics.

**Table 1.** Characteristics of self-reporting autistic adults (*n* = 1026), children with autism (*n* = 286) and their reporting parents (*n* = 286).

	Adults	Children	Parents
Age^a^, *M* (*SD*)	44.75 (13.72)	12.3 (2.84)	44.85 (5.44)
Educational level^b^			
Low	12.5%	–	7.4%
Middle	37.0%	–	32.5%
High	50.5%	–	60.1%
Autism traits^c^ (AQ), *M* (*SD*)	83.67 (10.90)	80.60 (11.17)	–
Gender			
Men	42.4%	78.0%	4.5%
Women	56.7%	22.0%	95.5%
Other	0.9%	–	–
IQ^d^			
<40	0.0%	0.7%	–
41–55	0.2%	6.5%	–
56–70	0.4%	8.2%	–
71–85	2.1%	11.8%	–
86–115	26.8%	41.6%	–
116–130	47.6%	27.2%	–
>130	23.0%	3.9%	–

SD: standard deviation.

a 1 missing value (0.1%) in the group of adults, 1 missing value (0.3%) in the children’s group and 5 missing values (2%) in parents’ data.

b 93 missing values (9%) in educational data of the adults and 15 missing values (5%) in parents’ educational data.

c 40 missing adult AQ data (4%) and 82 missing child AQ data (29%).

d 7 missing IQ data (0.7%) in the group of adults and 7 missing IQ data (2%) in the children’s group.

### Material

The topic of autism terminology preference was introduced as follows: ‘The questions below are about the portrayal of autism. Which label do you prefer when it comes to people with autism?’ Respondents could select one of the six terminology options that they most preferred. These options were subsequently labelled as PFL, IFL, or other/no clear preference. The options were originally presented in Dutch, but for this paper translated to English: (1) People with autism (PFL), (2) Autists/Autistics (IFL; in Dutch the term ‘autisten’ was used), (3) Autistic people (IFL), (4) Someone with Asperger’s, PDD-NOS or McDD (PFL), (5) None of the above (other), (6) Other, namely (recoded as PFL/IFL/other depending on the exact answer). If option 6 was chosen, individual answers were reclassified by two independent raters according to the category (PFL/IFL/other) they belonged to. Inter-rater reliability was excellent (kappa = .93).

*Autism traits* were measured using the abbreviated 28-item version of the Autism-Spectrum Quotient (AQ-Short). This is a questionnaire that was either self-rated or parent-rated, based on 28 statements that describe the social behaviours, interests and preferences of the person with autism (e.g. ‘I enjoy meeting new people’). Responses are rated on a four-point Likert-type scale ranging from 1 (definitely agree) to 4 (definitely disagree). Total AQ-Short score varies from 28 to 112, with higher scores indicating more autistic traits. The AQ-Short is highly correlated with the original 50-item AQ and has good psychometric properties (Hoekstra et al., 2011).

*Intellectual ability* was measured using self-/parent-reported IQ at one of the seven levels, ranging from IQ below 40 (severe intellectual disability) to IQ above 130 (gifted). IQ reports were either based on an IQ test performance prior to and independent of the current study (63% of adult self-reports; 86% of parent reports) or an estimation of the intellectual ability of the autistic person. Previous research has shown that proxy-reported IQ correlates highly with adaptive functioning (*r* = −0.71), providing preliminary evidence of the validity of this IQ measure (Werkman et al., 2020). In addition, in this study, we found overlap between people’s self-reported IQ levels and their educational levels. In the order from high to low IQ levels, we found that 65% (IQ > 130), 57% (IQ: 116–130), 31% (IQ: 86–115), 5% (IQ: 71–85), and 0% (IQ < 71) of the participants had obtained a high educational degree.

*Educational level* of autistic adults and parents was defined by their highest level of successfully completed education. Educational levels were coded high (e.g. university), middle (e.g. secondary vocational education) and low (e.g. pre-vocational secondary school), following the guidelines of Statistics Netherlands.

### Procedure

Data were acquired from the Netherlands Autism Register (NAR), a longitudinal database on children and adults with autism in the Netherlands. Respondents are either people with autism of 16 years and older reporting on themselves, or parents/legal representatives of children/adults with autism. Upon registration, respondents sign a digital informed consent form. After registration, respondents receive a yearly request via email to fill in an online questionnaire on autism-related topics. Data for this terminology study were collected in 2019. The NAR’s research has been evaluated and approved by the ethics committee of the Vrije Universiteit Amsterdam (VCWE 2020-041R1).

### Community involvement

A large-scale inventory of research priorities among NAR participants indicated a preference among adult autistic participants for more research about the portrayal and societal inclusion of autism. This study is a part of this research focus. NAR research is thus inspired by ideas from the autism and autistic community. The NAR also has autistic team members involved in all stages of research.

### Data analysis

First, we examined the proportion of people with a PFL/IFL/other autism terminology preference. With a Chi-square test we checked whether terminology preference differed for self-reporting autistic adults and parents. Multinomial logistic regression analyses were then used to predict autism terminology preference (PFL/IFL/other) for adults and parents separately. In the group of adults with autism, participants’ gender, age, intellectual ability, degree of autism traits (AQ) and educational level were entered as predictors. In the group of parents, parent’s age and educational level were entered as predictors, as well as child’s gender, intellectual ability and degree of autism traits (AQ). Significance level was set at .05.

## Results

Counter to our expectation, a majority of both self-reporting autistic adults and parents of a child with autism demonstrated a PFL preference (overall 71.4% preferred PFL). A small number of the open answers was coded ‘other’. The ‘other’ category included answers such as no clear preference for PFL or IFL. A PFL preference was more dominant in the group of parents (82.5%) compared to the adults with autism (68.3%, χ^2^(2) = 22.27, *p* < .001; Table 2), yet both groups showed a preference for PFL. Tables 3 and 4 show the characteristics of autistic adults and parents according to their terminology preference.

**Table 2.** Autism terminology preference.

	Person-first	Identity-first	Other
Autistic adults (*n* = 1026)	68.3% ‘People with autism’: 60.0% ‘Someone with Asperger’s, PDD-NOS or McDD’: 8.3%	22.7% ‘Autists/Autistics’: 16.9% ‘Autistic people’: 5.8%	9.0%
Parents (*n* = 286)	82.5% ‘People with autism’: 79.9% ‘Someone with Asperger’s, PDD-NOS or McDD’: 2.8%	11.9% ‘Autists/Autistics’: 6.6% ‘Autistic people’: 5.2%	5.6%

**Table 3.** Background characteristics of self-reporting adults with autism categorized according to their autism terminology preference.

Characteristic	Autism terminology preference			*F*/χ^2^
	Person-first (*n* = 701)	Identity-first (*n* = 233)	Other (*n* = 92)	
Age, *M* (*SD*)	45.59 (13.72)	42.51 (13.68)	44.00 (13.25)	5.60*
Autism traits (AQ), *M* (*SD*)	83.39 (10.88)	85.32 (10.95)	81.65 (10.51)	4.42*
Gender				9.56*
Men (*n* = 435)	44.5%	36.9%	40.2%	
Women (*n* = 582)	55.1%	60.9%	58.7%	
Other (*n* = 9)	0.4%	2.1%	1.1%	
IQ				21.83***
85 or lower (*n* = 27)	2.3%	3.0%	4.4%	
86–115 (*n* = 273)	31.0%	19.5%	13.2%	
116 or higher (*n* = 719)	66.7%	77.5%	82.4%	
Educational degree				10.24*
Low (*n* = 117)	12.8%	13.8%	7.4%	
Middle (*n* = 345)	37.4%	40.0%	25.9%	
High (*n* = 471)	49.8%	46.2%	66.7%	

SD: standard deviation.

* *p* < .05, ***p < .001.

**Table 4.** Background characteristics of children with autism and their parents categorized according to parents’ autism terminology preference.

	Autism terminology preference			*F*/χ^2^
	Person-first (*n* = 236)	Identity-first (*n* = 34)	Other (*n* = 16)	
Child’s age, *M* (*SD*)	12.37 (2.79)	12.44 (2.39)	11.40 (4.25)	0.91
Parent’s age, *M* (*SD*)	44.92 (5.52)	44.50 (5.28)	44.44 (4.81)	0.13
Parent’s educational level				4.00
Low	7.6%	9.7%	0.0%	
Middle	31.1%	32.3%	53.3%	
High	61.3%	58.1%	46.7%	
Child’s autism traits, *M* (*SD*)	80.35 (11.50)	82.65 (10.50)	80.20 (5.98)	0.44
Child’s gender				0.17
Boys (*n* = 223)	77.5%	79.4%	81.3%	
Girls (*n* = 63)	22.5%	20.6%	18.8%	
Child’s IQ				6.02
85 or lower (*n* = 76)	25.0%	42.4%	28.6%	
86–115 (*n* = 116)	44.0%	24.2%	42.9%	
116 or higher (*n* = 87)	31.0%	33.3%	28.6%	

SD: standard deviation.

A multinomial regression model predicting autism terminology preference (PFL/IFL/other with PFL as the reference category) in 886 self-reporting autistic participants was significant, χ^2^(14) = 46.40, *p* < 0.001, Nagelkerke *R*^2^ = 0.06, Cox & Snell *R*^2^ = 0.05. Even though there was an overall preference for PFL, younger adults with many autistic traits and a higher IQ were (relatively) more likely to prefer IFL compared to PFL (Table 5). No effects of gender or educational level were found. Adults with a higher IQ or high educational degree (vs middle educational degree) were more likely to prefer other terminology compared to PFL. No other associations were found.

**Table 5.** Multinomial regression model predicting autism terminology preference in adults with autism (with person-first language as the reference category).

	Predictor	*B* (*SE*)	OR (95% CI)	Wald	*p*
IFL vs PFL	Age	−0.02 (0.01)	0.98 (0.97–1.00)	6.92	.**01**
	Autism traits (AQ)	0.02 (0.01)	1.02 (1.00–1.03)	4.61	.**03**
	Intellectual ability (IQ)	0.39 (0.11)	1.48 (1.18–1.84)	11.93	.**001**
	Gender				
	Men vs women	−0.13 (0.18)	0.88 (0.62–1.25)	0.50	.48
	Other gender vs women	0.78 (0.83)	2.18 (0.43–11.12)	0.89	.35
	Educational level				
	Low vs High	0.29 (0.26)	1.34 (0.80–2.25)	1.25	.26
	Middle vs High	0.28 (0.19)	1.32 (0.92–1.90)	2.31	.13
Other vs PFL	Age	−0.01 (0.01)	0.99 (0.97–1.01)	1.69	.19
	Autism traits (AQ)	−0.02 (0.01)	0.99 (0.97–1.01)	1.77	.18
	Intellectual ability (IQ)	0.45 (0.17)	1.57 (1.12–2.20)	6.83	.**01**
	Gender				
	Men vs women	0.02 (0.26)	1.02 (0.61–1.71)	0.01	.93
	Other gender vs women	0.60 (1.19)	1.83 (0.18–18.67)	0.26	.61
	Educational level				
	Low vs High	−0.61 (0.47)	0.54 (0.22–1.34)	1.74	.19
	Middle vs High	−0.60 (0.29)	0.55 (0.31–0.97)	4.25	.**04**

IFL: identity-first language; PFL: person-first language; *SE*: standard error; OR: odds ratio; CI: confidence interval.

Significant *p*-values (*p* < .05) are in bold.

Within the group of parents with complete data (*n* = 198), the multinomial regression model predicting autism terminology preference was nonsignificant, χ^2^(14) = 9.37, *p* = 0.81, Nagelkerke *R*^2^ = 0.07, Cox & Snell *R*^2^ = 0.05. Thus, terminology preference of parents did neither depend on their educational level or age, nor on their child’s gender, AQ or IQ score.

## Discussion

Based on a large sample of Dutch-speaking autistic adults and parents of children with autism, we found an unexpected preference for PFL (‘person with autism’) over IFL (‘autistic person’). While this preference was more pronounced in the group of parents (82.5%), autistic adults preferred PFL as well (68.3%). Younger age, more autistic traits and a higher intellectual ability were identified as predictors of a preference for IFL compared to PFL in autistic adults. Within the group of parents, no predictors of terminology preference were found.

A clear PFL preference in adults with autism contrasts with an IFL preference previously reported in autistic adults in two empirical studies in English-speaking countries (Bury et al., 2020; Kenny et al., 2016). A first explanation for these contrasting results may be related to language differences. A previous study in a Spanish-speaking sample also revealed a PFL preference among non-autistic adults (Garcia-Molina, 2019). However, counter to the Spanish language, Dutch and English are both considered West Germanic languages that share basic syntactic rules (e.g. adjectives precede the noun), thus ruling out syntax as an explanation for different study outcomes. Another explanation for a PFL preference in our participants with autism may result from the derogatory use of some IFL options in the Dutch language. Indeed, ‘autist’ (IFL) was chosen by only a minority of the adults. However, as IFL options are also used as insults in the English language sometimes, this does not suffice to explain differences in study outcomes. Finally, cultural norms may have contributed to a PFL preference in our Dutch sample. In Dutch Calvinist tradition, emphasis is placed on modesty, soberness and conformity (Gordijn, 2010). An illustration of this is a well-known Dutch saying: ‘Act normally, that’s already crazy enough’. Whereas the neurodiversity movement, generally in favour of IFL, accepts and celebrates individual differences (Kapp et al., 2013), Dutch norms may dictate conformity instead. These cultural norms might make it more difficult for people with autism to stand out and express and embrace their unique autistic identity, which might include the use of IFL (Botha et al., 2020). Yet, as mentioned in the introduction, both proponents of IFL and PFL may regard autism as a vital part of their identity; therefore, ‘identity-first language’ may not be the most adequate term. Instead, we suggest that ‘autism-first language’ may be a better fitting alternative.

Sample and method differences may also explain the different outcomes of this and previous studies. Even though the Dutch self-reporting participants were quite similar to the English-speaking autistic samples in terms of age and gender, there were differences with regard to participant recruitment as well as the exact questions and answering options provided. Both Kenny et al. (2016) and Bury et al. (2020) used convenience samples, including recruitment via social media and online fora, potentially leading to more activistic participants with an IFL preference. In our study, participants of the Netherlands Autism Register were not specifically recruited for this terminology study, and many participants were enrolled years prior to the current study. Furthermore, in the Kenny et al. (2016) study, participants could select multiple terms from a list (unlike the present study) or only one term (similar to this study). However, not all listed terms were clearly PFL or IFL (e.g. autism spectrum disorder; autism spectrum condition), complicating a direct comparison with this study. Bury et al. (2020) asked participants to rate their preference for and offensiveness of six different terms. A separate evaluation of each term is likely to offer a more nuanced picture, highlighting the different sentiments that each term evokes. For instance, while ‘autistic’ was the most preferred term by 38%, it was also the least preferred term by 28%.

The preference for PFL in this study was stronger among parents compared to the adults with autism, and was unrelated to parents’ and children’s demographic characteristics. A restriction of range may have weakened possible associations with parent’s age, as the youngest and oldest parent in our study were, respectively, 29 and 58 years old (in contrast to the wide age range of self-reporting autistic adults: 16–84 years). The stronger PFL preference of parents could be related to how professionals (usually PFL preference; Kenny et al., 2016) or Facebook pages (usually PFL preference; Abel et al., 2019) commonly communicate about autism to parents. Also, online autistic self-advocates (usually IFL preference; Sabatello, 2019; Shakes & Cashin, 2020; Thibault, 2014) may mostly reach other autistic people rather than their family members.

Among our younger adult participants, there was a *relatively* stronger preference for IFL (29.5% of 16- to 30-year-olds preferred IFL compared to 21.3% of participants older than 30 years). Younger adults may be more actively involved in the social media discourse related to neurodiversity compared to older adults. Awareness of the neurodiversity movement has previously been associated with an IFL preference (Kapp et al., 2013). The same argument – being more actively involved in social media and online fora – may also explain why individuals with higher IQ’s showed a relatively stronger preference for IFL, although a majority still preferred PFL (see also Table 3). Alternatively, autistic individuals with higher IQ’s may experience a large(r) discrepancy between their own talents and abilities, on one hand, and society’s expectations and treatment on the other. Indeed, in a systematic review of experienced stigma, Han et al. (2022) conclude there is a tension between societal perceptions and self-perceptions of autistic individuals with an (above) average intellectual ability. This tension may cause some people to conceal their autism, whereas it motivates others to open up and take pride in their autism (Han et al., 2022). In line with findings from Bury et al. (2020), individuals with more self-reported autistic traits also had a relatively stronger IFL preference. It could be that individuals with more autistic traits are more often confronted with questions and stigma about autism, possibly fortifying an autism-related identity and an IFL preference. There may also be a methodological explanation for the found association: Individuals with an IFL preference and an identity centred on autism may be more inclined to endorse autistic traits on a questionnaire. Finally, we did not find an association between gender and autism terminology preference. Corresponding with findings by Bury et al. (2020), a majority (56%) of autistic individuals who indicated an ‘other’ gender expressed an IFL preference, and they were twice as likely to prefer IFL over PFL than autistic women (OR = 2.18). However, this group was unfortunately too small to have a statistically significant effect. Belonging to multiple minorities might re-enforce an IFL preference, but this remains speculation for now.

A limitation of this study is that the number and type of options for participants to choose from were limited and not identical to the options provided in previous studies. This may have influenced the results. In hindsight, we would have liked to include the option ‘person on the autism spectrum’, as both Kenny et al. (2016) and Bury et al. (2020) demonstrated that this term was reasonably accepted by various stakeholder groups. However, including this (PFL) option may have produced an even stronger PFL preference. Second, until 2020, the Netherlands Autism Register (NAR) almost exclusively used PFL on the website, thus possibly attracting mostly people with a PFL preference. Third, the phrasing of the question itself (‘Which label do you prefer when it comes to people with autism?’) may have promoted a PFL preference. Finally, with regard to predictors of IFL/PFL preference, we recognize that very little variance could be explained by age, autistic traits and intellectual ability. For future studies, we recommend the use of more objective tests of intelligence and autistic traits to elaborate on and replicate these findings. Other potential variables of interest for further study are experiences of stigmatization and discrimination as well as internalized stigma, as IFL may sometimes be a response to stigma (Han et al., 2022).

The key message of our study findings is that language and culture may impact the preference for identity/autism-first or person-first language, as we noticed a stronger person-first language preference among our Dutch participants compared to an identity/autism-first language preference in English-speaking autistic participants in prior studies. Additional research, both inside and outside English-speaking countries, is needed to get a better grasp of the term preferences of autistic people. The choice for PFL or IFL should first be guided by what the majority of autistic individuals, their families and professionals prefer. At the same time, considering the large differences in terminology preferences both within and between groups (Bury et al., 2020; Kenny et al., 2016), we advise to check a person’s own preference in a one-on-one interaction. Moreover, the potentially stigmatizing and harming effects of PFL and IFL should be examined more closely, possibly in an experimental design. If either PFL or IFL has a strong negative effect on a small group of people, the majority argument may no longer hold. For now, we suggest to use a mix of both PFL and IFL in order to cover all people’s preferences. As terminology will continue to evolve over time, research on changing preferences is warranted.
